# One-Year Readmissions Following Total Joint Arthroplasty May Be Associated With Failure to Achieve the Minimal Clinically Important Difference of Patient-Reported Outcomes Measurement Information System Physical, Mental, and Physical-Short Form-10a

**DOI:** 10.7759/cureus.32181

**Published:** 2022-12-04

**Authors:** Akhil Katakam, Wayne Cohen-Levy, Shayan Hosseinzadeh, Isabella Florissi, Yhan Colon Iban, Tyler J Humphrey, Amy Z Blackburn, Christopher M Melnic, Antonia Chen, Todd O'Brien, Charles Bragdon, Hany S Bedair

**Affiliations:** 1 Orthopaedic Surgery, Rutgers Robert Wood Johnson Medical School, Boston, USA; 2 Orthopaedic Surgery, Case Western Reserve University, Cleveland, USA; 3 Orthopaedic Surgery, Massachusetts General Hospital, Boston, USA; 4 Orthopaedic Surgery, Brigham and Women's Hospital, Boston, USA

**Keywords:** promis-sf10a, minimal clinically important difference, readmission, total joint arthroplasty, patient reported outcome measures

## Abstract

The primary aims of our study were to determine if hospital readmissions within one year following primary total joint arthroplasty (TJA) and their relative timing influence patients’ ability to achieve the two-year Patient-Reported Outcomes Measurement Information System (PROMIS) physical, PROMIS mental, and PROMIS Physical-Function-Short-Form-10a (SF-10a) minimal clinically important difference (MCID). This is a retrospective study conducted using data from a multi-institutional, arthroplasty registry. Only patients with paired patient-reported outcome measure (PROM) assessments (preoperatively and two years postoperatively) were included. Five separate readmission cohorts were formed: (1) any-cause readmission within one year, (2) any-cause readmission within 90 days, (3) non-index-surgery-related readmission within 90 days, (4) index-surgery-related readmission within one year, and (5) index-surgery-related readmission within 90 days. A propensity score match was used to match each of the patients to one of the 972 patients (1:1 basis) in the non-readmission group. The association between failure to achieve each of the three two-year MCIDs and Readmission status was analyzed using logistic regression. We found that all readmissions within one year and index-surgery-related readmissions within one year resulted in an increased risk of failure to achieve the two-year MCID across all three collected PROMs. Index surgery-related readmissions within 90 days (OR 3.24; 95% CI 1.05-11.05; p=0.048) sustained significantly different rates of two-year PROMIS physical MCID achievement compared to matched controls. Postoperative complications requiring readmission, particularly those related to the joint arthroplasty and those within 90 days of index surgery, significantly impact the ability to achieve the two-year MCID of PROMs.

## Introduction

The increasing demand and cost for arthroplasty care have necessitated a need to monitor outcomes [1-3]. Metrics such as infection rates, re-operation rates, and hospital readmission rates are measures of postoperative outcomes, but are relatively infrequent events [4]. Patient-reported outcome measures (PROMs), on the other hand, allow patients to categorize a subjective analysis of their health state on a variety of dimensions, including general health, pain, mental health, and overall physical function. The Patient-Reported Outcomes Measurement Information System (PROMIS), developed by the National Institute of Health, is a 10-question survey that measures both the domains of physical function (PROMIS physical) and mental health (PROMIS mental) and is useful in a variety of diverse treatments and procedures, including total joint arthroplasty (TJA) [5-9]. There is also an abbreviated version, the Physical Function Short Form 10a (SF-10a), which also consists of 10 questions and determines a patient’s physical function level. To aid in the interpretation of raw PROM scores, various PROM metrics have been developed, such as the Patient Acceptable Symptom State (PASS) and the Minimal Clinically Important Difference (MCID) [10-11]. These metrics evaluate the pre- to postoperative difference in PROM scores that are deemed clinically significant for the average patient [12-15].

There is a growing understanding of how adverse surgical events, such as periprosthetic infections, revisions, and hospital readmissions may affect patient satisfaction, PROM scores, and the ability to achieve MCID. While some of these events are discrete and easy to categorize, hospital readmissions are quite diverse and require a deeper analysis due to their frequency and lack of uniformity. For example, readmission for a periprosthetic joint infection or a myocardial infarction constitutes two vastly different pathophysiologic events and therefore can impact postoperative outcomes in varying ways [16-18]. Furthermore, many analyses on readmissions focus on those events within the first 90-day postoperative period, which may be driven by insurer-based definitions of the “global” period of care. There is limited work reporting on readmission beyond the first 90 days and its implications for patient outcomes.

The primary aim of this study was to investigate whether hospital readmission within one year following primary TJA and its timing relative to the index surgery influenced patients’ ability to achieve the two-year PROMIS physical, PROMIS mental, and SF-10a MCID.

## Materials and methods

Level of Evidence III. This retrospective study was conducted with Institutional Review Board approval using data from a regional, multi-institutional, arthroplasty registry. This registry is populated from the electronic medical records of patients treated in a healthcare network comprised of seven hospitals. All cases of primary total hip arthroplasty (THA) and total knee arthroplasty (TKA) performed since 2016 within this hospital network are captured in the registry as well as all postoperative readmissions with the same network, with an approximate 10% lost-to-follow-up rate within one year of surgery [19]. The PROMIS physical, PROMIS mental, and SF-10a scores are collected preoperatively and at yearly postoperative intervals. Only patients with paired PROM assessments (preoperatively and two years postoperatively) were included in this analysis. In order to maximize the number of patients for analysis and to account for inherent variations in the timing of patient follow-up visits, preoperative scores were defined as those captured within six months prior to the index procedure and the two-year postoperative period as those collected between 1.5 and 2.5 years from the index surgery.

A registry query for all primary THA and primary TKA patients with complete preoperative and two-year postoperative PROM score sets resulted in 1,302 patients. Variables collected for each patient included: demographics (age at index surgery, sex, body mass index [BMI]), Charlson Comorbidity Index (CCI), preoperative diagnoses (myocardial infarction, cancer, cerebrovascular accident, diabetes mellitus, hypertension, or congestive heart failure), discharge disposition, length of stay, and identity of the institution where the THA or TKA was performed. PROM scores for these patient populations were used to derive the three distinct MCIDs at the two-year postoperative timepoint using the distribution-based method, which defines the MCID as one-half of the standard deviation of the preoperative to postoperative change in each PROM for the entire population [20-22]. Of the 1,302 patients who completed preoperative and two-year PROM assessments, two-year MCIDs of 4.02, 4.46, and 6.19 for the PROMIS physical, PROMIS mental, and SF-10a, respectively, were determined.

A rigorous chart review was performed to classify the primary reason for the initial hospital readmission. Patients were then categorized into those having any readmission within one year of the index TJA and those having no readmissions. Patients undergoing subsequent elective procedures within 90 days of index TJA (n=34) were excluded from further analysis because of the inherent difficulty in attributing the complication readmission as related to the first elective procedure or the second elective procedure. Patients with multiple readmissions were categorized by only the readmission related to index TJA. If none of the readmissions pertained to index TJA, only the first readmission was categorized. With respect to timing and indication, readmission events were classified according to timing as early (within 90 days of index surgery) or any (0-365 days) and the reason for the readmission (surgical or other). The index-surgery-related readmissions were further classified according to the chief complaint identified by the consulted orthopaedic surgeon in the discharge summary. For non-index-surgery-related readmissions, the chief complaint as identified in the patient’s discharge summary was recorded and further classified by organ system. A complete list of readmission events is included in Appendix A. Readmission events are further stratified by readmission group in Appendix B.

Five separate readmission cohorts were formed: (1) any-cause readmission within one year (n=296), (2) any-cause readmission within 90 days (102), (3) non-index surgery-related readmission within 90 days (n=58), (4) index surgery related readmission within one year (n=90), and (5) index-surgery related readmission within 90 days (n=44).

Statistical analysis

A propensity score match was used to match each patient in the five subgroups to one of the 972 patients (1:1 basis) in the non-readmission group. A separate propensity score match was performed for each PROM. Patients were matched on age, BMI, history of myocardial infarction, cancer, or cerebrovascular accident, CCI, congestive heart failure, type II diabetes, discharge disposition, hospital, length of stay, sex, and joint. Chi-squared test of independence and student’s t-test were used to compare categorical and continuous demographic variables respectively, between all patients (unmatched) as well as the matched cohorts. For continuous variables used in the propensity score matching, matching was performed for values within 0.5 standard deviations of the mean of the variable. For each patient in the non-readmission and readmission cohorts, the change from preoperative to two-year postoperative PROM assessments was calculated and used to determine whether that patient achieved the two-year MCID. The three primary outcomes of interest were failure to achieve PROMIS physical, PROMIS mental, and SF-10a MCIDs at two years. Readmission status was interpreted as a binary variable in regression analysis. For each matched readmission cohort, every candidate predictor variable was included in multivariable logistic regression so as to not omit potential interactions. For only the variables that were significantly associated (p<0.05) with the outcome of interest after regression analysis, odds ratios (OR) and corresponding 95% confidence intervals (CI) were reported. All statistical analyses were performed using R (The R Foundation, Vienna, Austria) and RStudio (RStudio, Boston, MA, USA).

## Results

Achieving MCID and readmissions (matched cohorts)

For patients with any readmission (all-cause) within one year (n=296), 133 (44.9%), 194 (65.5%), and 157 (53.0%) patients failed to achieve the two-year PROMIS physical, PROMIS mental, and SF-10a MCIDs, respectively (Table 1). Following multivariable regression on matched cohorts, readmission within one year was a significant risk factor for failure to achieve two-year PROMIS physical (OR 1.43; 95% CI 1.01-2.03; p=0.042), PROMIS mental (OR 1.46; 95% CI 1.02-2.09; p=0.042), and SF-10a (OR 1.44; 95% CI 1.01-2.05; p=0.043) MCID following TJA (Table 2).

**Table 1:** Patient Demographics and Primary Patient-Reported Outcome Measures Scores of All-Cause Readmissions Within One-Year Cohort CVA: cerebrovascular accident; CCI: Charlson Comorbidity Index; CHF: Congestive Heart Failure; SNF: Skilled Nursing Facility; BWH: Brigham and Women's Hospital; FH: Faulkner Hospital; MGH: Massachusetts General Hospital; NSMC: North Shore Medical System ; NWH: Newton-Wellesley Hospital; PROMIS: Patient-Reported Outcomes Measurement Information System; MCID: Minimal Clinically Important Difference; SF-10a: Short form-10a * Statistically Significant

	Unmatched			Matched		
Variables	No Readmissions (n=972)	Readmission in 1 Year (n=296)	P-value	No Readmissions (n=296)	Readmission In 1 Year (n=296)	P-value
Myocardial Infarction	13	4	0.985	1	4	0.178
Age	64.9	66.0	0.080	65.9	66.0	0.860
Body Mass Index	29.8	30.6	0.033*	31.1	30.6	0.346
Cancer	164	66	0.040*	63	66	0.765
CVA	24	13	0.085	8	13	0.267
CCI	0.9	1.1	0.209	1.1	1.1	0.749
CHF	16	9	0.131	10	9	0.816
Type II Diabetes	84	33	0.192	35	33	0.797
Discharge						
Home or Self Care	41	16	0.388	13	16	0.568
Home Healthcare	787	211	<0.001*	219	211	0.461
Rehabilitation Facility	31	14	0.210	13	14	0.844
SNF	113	55	0.002*	51	55	0.668
Hospital						
BWH	272	82	0.925	85	82	0.784
FH	129	45	0.398	44	45	0.908
MGH	340	102	0.869	102	102	1.000
NSMC	61	21	0.616	24	21	0.642
NWH	170	46	0.435	41	46	0.562
Length of Stay	2.4	2.7	0.001	2.7	2.7	0.927
Male Sex	409	129	0.116	129	129	1.000
Total Knee Arthroplasty	531	182	0.037*	197	182	0.199
Preoperative PROMIS physical	42.3	41.3	0.054	41.3	41.3	0.997
Failure to Achieve 2-year PROMIS physical MCID	367	133	0.027*	111	133	0.066
Preoperative PROMIS mental	50.8	49.4	0.034	49.9	49.4	0.535
Failure to Achieve 2-year PROMIS mental MCID	591	194	0.142	175	194	0.107
Preoperative SF-10a	35.4	36.2	0.223	34.5	36.2	0.023
Failure to Achieve 2-year SF-10a MCID	410	157	0.001	130	157	<0.001

**Table 2:** Multivariable Regression of Matched One-Year Readmission Cohort to Predict Failure to Achieve A) Two-Year PROMIS Physical, B) Two-Year PROMIS Mental, and C) SF-10a MCID With Significant Variables Reported (Matched) PROMIS: Patient-Reported Outcomes Measurement Information System; MCID: Minimal Clinically Important Difference; BMI: Body Mass Index; SF-10a: Short form-10a

Variable	Odds Ratio	95% Confidence Interval	P-value
Readmission within 1 year	1.43	1.01-2.03	0.042
Cerebrovascular Accident	2.75	1.04-7.69	0.045
Diabetes Mellitus	1.99	1.09-3.69	0.027
Length of Stay	1.22	1.07-1.41	0.005
Preoperative PROMIS physical	1.08	1.05-1.10	<0.001

Variable	Odds Ratio	95% Confidence Interval	P-value
Readmission within 1 year	1.46	1.02-2.09	0.042
Preoperative PROMIS mental	1.08	1.06-1.11	<0.001

Variable	Odds Ratio	95% Confidence Interval	P-value
Readmission within 1 year	1.44	1.01-2.05	0.043
BMI	1.05	1.01-1.08	0.005
Cerebrovascular Accident	3.13	1.12-9.84	0.036
Diabetes Mellitus	1.95	1.04-3.73	0.039
Total Knee Arthroplasty	1.56	1.07-2.29	0.021
Preoperative SF-10a	1.09	1.06-1.12	<0.001

MCID and index surgery-related readmissions

Index surgery-related readmissions within one year were a significant risk of failure to achieve the two-year PROMIS physical (OR 1.93; 95% CI 1.02-3.72; p=0.045), PROMIS mental (OR 2.01; 95% CI 1.02-4.04; p=0.047), and SF-10a (OR 2.04; 95% CI 1.04-4.06; p=0.039) MCID compared to matched non-readmission cohort (Table 3). Table 4 compares all demographics between matched and unmatched cohorts for index-surgery-related complications within one year of index TJA.

**Table 3:** Multivariable Regression of Matched One-Year Orthopaedic-Related Readmission Cohort to Predict Failure to Achieve A) Two-Year PROMIS-10 Physical, B) Two-Year PROMIS-10 Mental Health, and C) PROMIS Physical Function – Short Form 10a MCID With Signific PROMIS: Patient-Reported Outcomes Measurement Information System; MCID: Minimal Clinically Important Difference; SF-10a: Short form-10a

Variable	Odds Ratio	95% Confidence Interval	P-value
Index Surgery-related Readmission within 1 Year	1.93	1.02-3.72	0.045
Preoperative PROMIS physical	1.09	1.04-1.15	0.001

Variable	Odds Ratio	95% Confidence Interval	P-value
Index Surgery-related Readmission within 1 Year	2.01	1.02-4.04	0.047
Preoperative PROMIS mental	1.08	1.03-1.13	0.001

Variable	Odds Ratio	95% Confidence Interval	P-value
Index Surgery-related Readmission within 1 Year	2.04	1.04-4.06	0.039
Preoperative SF-10a	1.13	1.06-1.21	0.001

**Table 4:** Patient Demographics of Index Surgery-Related Readmission Within One-Year Cohort CVA: Cerebrovascular Accident; CCI: Charlson Comorbidity Index; CHF: Congestive Heart Failure; SNF: Skilled Nursing Facility; BWH: Brigham and Women's Hospital; FH: Faulkner Hospital; MGH: Massachusetts General Hospital; NSMC: North Shore Medical Center; NWH: Newton-Wellesley Hospital; PROMIS: Patient-Reported Outcomes Measurement Information System; MCID: Minimal Clinically Important Difference; SF-10a: Short form 10a

	Unmatched			Matched		
Variables	No Readmissions (n=972)	Index Surgery Related Readmission in 1 Year (n=90)	P-value	No Readmissions (n=90)	Index Surgery Related Readmission in 1 Year (n=90)	P-value
Myocardial Infarction	13	1	0.857	0	1	0.316
Age	64.9	65.4	0.670	66.2	65.4	0.505
Body Mass Index	29.8	31.1	0.041*	30.4	31.1	0.404
Cancer	164	16	0.827	17	16	0.847
CVA	24	5	0.086	5	5	1.000
CCI	0.9	0.8	0.164	0.8	0.8	0.943
CHF	16	1	0.699	0	1	0.316
Type II Diabetes	84	5	0.312	3	5	0.469
Discharge						
Home or Self Care	41	5	0.551	3	5	0.469
Home Healthcare	787	70	0.463	68	70	0.724
Rehabilitation Facility	31	5	0.235	4	5	0.732
SNF	113	10	0.884	15	10	0.281
Hospital						
BWH	272	32	0.396	33	32	0.890
FH	129	13	0.754	16	13	0.543
MGH	340	28	0.461	29	28	0.873
NSMC	61	8	0.336	7	8	0.787
NWH	170	9	0.069	5	9	0.266
Length of Stay	2.4	2.7	0.028*	2.8	2.7	0.841
Male Sex	409	36	0.702	30	36	0.353
Total Knee Arthroplasty	531	63	0.005*	64	63	0.870
Preoperative PROMIS physical	42.3	40.8	0.084	40.3	40.8	0.656
Failure to Achieve 2-year PROMIS physical MCID	367	46	0.013*	33	46	0.007*
Preoperative PROMIS mental	50.8	49.5	0.225	49.1	49.5	0.754
Failure to Achieve 2-year PROMIS mental MCID	591	60	0.275	47	60	0.048
Preoperative SF-10a	35.4	35.8	0.692	34.2	35.8	0.234
Failure to Achieve 2-year SF-10a MCID	410	52	0.004	39	52	0.053

MCID and readmissions within 90 days

Index surgery-related readmissions within the 90 days cohort also sustained significantly different rates of two-year PROMIS physical (OR 3.24; 95% CI 1.05-11.05; p=0.048) MCID achievement compared to matched controls (Table 5). Table 6 compares the demographic variables collected between unmatched and matched cohorts for index surgery-related readmissions within 90 days of the index TJA group. However, all readmissions within 90 days were only found to be predictive of failure to achieve the two-year PROMIS mental (OR 2.04; 95% 1.07-3.97; p=0.033) MCID, but not the PROMIS physical or SF-10a MCID (Table 7). Appendix C compares the matched and unmatched cohorts of the all-cause readmission group within 90 days. Patients in the non-index surgery-related readmission cohort within 90 days (n=58) fared similarly to matched controls for failure to achieve two-year MCID, with 28 (48.3%) and 31 (53.4%) patients failing to achieve the MCID in the matched non-readmission and non-index surgery-related within 90-day readmission cohorts, respectively (Appendix D). Preoperative PROM score was the only variable consistently predictive of MCID failure amongst all three PROMs (Appendix E).

**Table 5:** Multivariable Regression of Matched 90-Day Index Surgery-Related Readmission Cohort to Predict Failure to Achieve A) Two-Year PROMIS-10 Physical, B) Two-Year PROMIS-10 Mental Health, and C) PROMIS Physical Function – Short Form 10a MCID With Signific PROMIS: Patient-Reported Outcomes Measurement Information System; MCID: Minimal Clinically Important Difference; SF-10a: Short form 10a

Variable	Odds Ratio	95% Confidence Interval	P-value
Index Surgery-related Readmission within 90 days	3.24	1.05-11.05	0.048
Type II Diabetes	1.52	1.23-1.69	0.027
Length of Stay	2.82	1.41-6.44	0.006
Male Sex	5.76	1.56-26.49	0.014
Preoperative PROMIS physical	1.09	1.04-1.15	0.001
Variable	Odds Ratio	95% Confidence Interval	P-value
Preoperative PROMIS mental	1.13	1.04-1.26	0.0126
Variable	Odds Ratio	95% Confidence Interval	P-value
Length of Stay	2.88	1.38-7.04	0.01
Male Sex	9.52	2.29-52.75	0.004
Preoperative SF-10a	1.17	1.07-1.34	0.007

**Table 6:** Patient Demographics of Index Surgery-Related Readmission Within 90-Days Cohort CVA: Cerebrovascular accident; CCI: Charlson Comorbidity Index; CHF: Congestive Heart Failure; SNF: Skilled nursing facility; BWH: Brigham and Women's Hospital; FH: Faulkner Hospital; MGH: Massachusetts General Hospital; NSMC: North Shore Medical Center; NWH: Newton-Wellesley Hospital; PROMIS: Patient-Reported Outcomes Measurement Information System; MCID: Minimal Clinically Information System; SF-10a: Short Form-10a

	Unmatched			Matched		
Variables	No Readmissions (n=972)	Index Surgery Related Readmission in 90 days (n=44)	P-value	No Readmissions (n=44)	Index Surgery Related Readmission in 90 days (n=44)	P-value
Myocardial Infarction	13	1	0.603	2	1	0.557
Age	64.9	66.7	0.196	66.9	66.7	0.928
Body Mass Index	29.8	30.4	0.534	29.8	30.4	0.603
Cancer	164	6	0.574	8	6	0.560
CVA	24	4	0.009*	6	4	0.502
CCI	0.9	0.7	0.161	0.8	0.7	0.578
CHF	16	0	0.391	0	0	1.000
Type II Diabetes	84	3	0.672	0	3	0.078
Discharge						
Home or Self Care	41	3	0.407	3	3	1.000
Home Healthcare	787	35	0.814	36	35	0.787
Rehabilitation Facility	31	3	0.190	4	3	0.694
SNF	113	3	0.327	1	3	0.306
Hospital						
BWH	272	14	0.580	16	14	0.653
FH	129	8	0.351	5	8	0.367
MGH	340	14	0.667	14	14	1.000
NSMC	61	3	0.885	3	3	1.000
NWH	170	5	0.293	6	5	0.747
Length of Stay	2.4	2.5	0.690	2.6	2.5	0.643
Male Sex	409	19	0.885	23	19	0.393
Total Knee Arthroplasty	531	31	0.039*	28	31	0.496
Preoperative PROMIS physical	42.3	41.5	0.509	42.4	41.5	0.593
Failure to Achieve 2-year PROMIS physical MCID	367	19	0.468	15	19	0.381
Preoperative PROMIS mental	50.8	50.1	0.620	52.2	50.1	0.240
Failure to Achieve 2-year PROMIS mental MCID	591	31	0.199	32	31	0.813
Preoperative SF-10a	35.4	35.2	0.876	33.2	35.2	0.419
Failure to Achieve 2-year SF-10a MCID	410	22	0.305	15	22	0.131

**Table 7:** Multivariable Regression of Matched All-Cause 90-Day Readmission Cohort to Predict Failure to Achieve A) Two-Year PROMIS-10 Physical, B) Two-Year PROMIS-10 Mental Health, and C) PROMIS Physical Function – Short Form 10a MCID With Significant Variable PROMIS: Patient-Reported Outcomes Measurement Information System; MCID: Minimal Clinically Important Difference; SF-10a: Short form-10a * Statistically significant

Variable	Odds Ratio	95% Confidence Interval	P-value
Length of Stay	1.84	1.30-2.71	0.001
Preoperative PROMIS physical	1.1	1.05-1.16	<0.001*
Variable	Odds Ratio	95% Confidence Interval	P-value
All-Cause Readmissions within 90 days	2.04	1.07-3.97	0.033
Preoperative PROMIS mental	1.09	1.04-1.14	<0.001*
Variable	Odds Ratio	95% Confidence Interval	P-value
Diabetes Mellitus	12.17	1.61-172.27	0.03
Preoperative SF-10a	1.14	1.07-1.22	<0.001*

Stratification of readmissions

Analysis of the all-cause within one-year readmission cohort (n=296) revealed index surgery-related complications were the leading cause of readmissions (n=90; 30.4%); gastrointestinal (n=47; 15.9%), cardiac (n=39; 13.2%), and orthopaedic complications not related to the index surgery (n=32; 10.8%). A complete list of complications stratified by readmission group is included in Appendix E. Infection-related complications (n=27; 30.0%) were the leading cause of index surgery-related admissions; lysis of adhesions following TKA (n=24; 26.7%) was the second reason for index surgery-related admissions.

## Discussion

As PROMs continue to enhance patient care by incorporating a patient’s subjective appraisal of a surgical procedure, metrics such as the MCID may help in interpreting the clinical significance of these outcomes. In investigating the association between readmissions and achievement of MCID, the authors found that patients with any hospital readmission within one year of TJA were less likely to achieve two-year MCID across PROMIS physical, PROMIS mental, and SF-10a compared to patients without a readmission event. It is important to note that these lower patient-reported outcomes were driven primarily by index surgery-related readmissions as opposed to readmissions for non-index surgery-related reasons. Furthermore, after stratifying by time, index surgery-related readmissions within 90 days of TJA were more strongly associated with failure to achieve PROMIS physical MCID as compared to matched controls. These findings can provide guidance for physicians when managing patient expectations and when providing postoperative care following TJA. To the authors’ knowledge, the current study is the first to distinguish between readmissions related to the arthroplasty procedure and those unrelated as it pertains to impact on three commonly used PROMs. The data supports the notion that readmissions following TJA are heterogeneous and can impact the ultimate outcome differently based on the indication for readmission and time relative to arthroplasty. It also provides reassurance to surgeons when asked about the impact of non-index surgery-related readmissions on ultimate recovery after TJA.

Previous studies have focused on pre-operative factors that can predict satisfaction following joint replacement [23]. Recent efforts to utilize machine learning algorithms to predict those patients who would achieve MCID pre-operatively produced models with poor-to-good accuracy [24]. These metrics, however, may be influenced by adverse surgical events such as hospital readmissions. The results herein offer some explanation for the performance of the algorithm as postoperative events can impact satisfaction and the ability to achieve MCID. Previous authors have established that readmissions can influence patient satisfaction and subjective outcome following TJA [25-26]. Bourne et al. noted that patients who had postoperative complications requiring readmission after primary TKA were 1.9 times more likely to be dissatisfied with their outcome [25]. Similarly, Friebel et al. were able to demonstrate improvements in functional and quality of life scores with reductions in readmission rates [26]. The current results support these previous findings as all-cause readmissions within one year of index surgery were associated with a lower rate of achieving MCID in the study cohort.

Beyond the actual measures, it is also critical to consider the time point at which these measurements are taken. Previous evidence has suggested that TJA patients continue to experience gains for up to one year after TJA [27]. However, the largest improvements in patient-reported outcome scores have been observed to occur within the first three months of surgery with smaller-scale improvements at six and 12 months [28]. This may explain the strong negative predictive value of complications that occurred strictly within 90 days of surgery as compared to complications occurring between postoperative days 0 and 365. Our results align with those of Neuprez et al., who were able to demonstrate that THA patients with early complications were three times less likely to report WOMAC scores that achieved MCID [28]. Interestingly, early complications were not similarly predictive in TKA patients [28]. Patient-reported complications within three and six months have also been shown to be predictive of lower functional and quality of life scores at those same time points [29]. Similarly, complications within one year of surgery have previously been shown to negatively impact the likelihood of achieving a minimally important difference in five-year WOMAC scores in TKA patients but not THA patients [28].

Conceptually, it is logical to infer that any interruption in a patients’ rehabilitation caused by a complication necessitating readmission during the acute postoperative period may impede them from achieving critical milestones, thus compromising the ultimate function of the joint. Similarly, the majority of readmissions unrelated to the index procedure may not be predictive of the patients’ ability to achieve MCID, with the rarer exceptions of more debilitating reasons for readmission such as unrelated lower extremity fractures, stroke with motor deficits, and intensive care unit admissions. Conversely, those readmissions occurring after 90 days are less impactful as the majority of the functional gains and recovery may have already been achieved and the patient has had an opportunity to experience the benefits of their replaced joint thus buoying their subjective assessment of the surgery.

This study, however, is not without limitations and its results should be interpreted within the context of its strengths and weaknesses. Given its retrospective nature, the current study is limited in identifying additional potentially confounding variables that were not collected in the registry. Additionally, readmissions to facilities outside of the institutional network were unable to be included and could impact the analysis as a source of measurement bias. It is worth noting that the authors’ institution does include a multitude of community and tertiary referral hospitals in the area, so the effects of uncaptured readmissions were theoretically minimized. Furthermore, loss-to-follow-up is a limitation inherent to any PROM analysis and this study is no exception. However, the arthroplasty registry utilized reports an approximate 10% lost-to-follow-up rate within one postoperatively, thus helping to mitigate such biases [19]. Another possible source of selection bias stems from only using preoperative and two-year postoperative PROMs; it is possible that different findings may result from PROMs completed longer after index TJAs.

In this study, we derived our own MCID values using a distribution-based method as opposed to using an anchor-based method given the retrospective nature of our study and lack of anchor questionnaire available [30]. The distribution has been used in numerous studies evaluating PROMs in TJA. We found that our distribution-based calculation of the MCID of PROMIS Physical, Mental and SF-10a were similar to previously described calculations [31-33]. We also chose to calculate a combined MCID of the selected PROMs for TJA, pooling the data of THA and TKA. As THA and TKA are different procedures, it is possible that the MCID of our selected PROMs could differ between the procedures, although previous studies have chosen to combine THA and TKA to calculate a TJA MCID as the values of the MCID for the individual procedure are similar [33].

Additionally, the current study includes patients from a variety of surgeons at multiple hospitals lending credence to the applicability of the results. Another possible limitation involves the omission of one-year postoperative PROM scores when assessing readmissions that occurred in the first postoperative year. Only the two-year postoperative period was assessed as it would account for time-dependent completion of the postoperative assessment and allow for an adequate period of recovery prior to assessment completion. We also chose to classify patients with multiple readmissions according to the readmission related to index TJA, which may be a source of bias as patients with multiple readmissions are expected to have worse outcomes. The number of patients included, and the two-year follow-up scores should be considered strengths of this study.

## Conclusions

In conclusion, postoperative complications requiring readmission, particularly those related to joint arthroplasty, significantly impact patient-reported outcomes and especially the ability to achieve the two-year MCID. Moreover, complications within 90 days are more impactful than complications that occur later on during the first postoperative year. This information is important as measures to mitigate index surgery-related complications and readmissions will have a significant impact in improving the proportion of patients achieving MCID on PROMs. 

## Appendices

Appendix A

**Table 8:** Distribution of All Readmission Events by Health Service Category ENT: Ear Nose and Throat; GI: Gastroenterology

Readmission Type	Frequency
Cardiology	39
Dermatology	11
Endocrine	2
ENT	7
GI	47
Immunology	10
Infectious Disease	7
Neurology	10
Ophthalmology	4
Orthopaedic (not related to index surgery)	32
Index Surgery Related	90
Psychiatry	3
Pulmonary	9
Renal/urology	21
Reproductive	4

Appendix B

**Table 9:** All-Cause Readmissions Within One Year (n=296), Stratified by Specific Cause for Readmission ENT: Ear Nose and Throat; GERD: Gastroesophageal Reflux Disease; TKA: Total Knee Arthroplasty; I&D: Irrigation and Debridement; COPD: Chronic obstructive pulmonary disease

Readmission Event	Frequency
Cardiology	39
Acute Chest Syndrome	1
Acute Myocardial Infarction	5
Aortic Dissection	1
Atrial Fibrillation	3
Chest Pain	5
Congestive Heart Failure	5
Coronary Artery Disease	2
Deep vein thrombosis	4
Palpitations	2
Peripheral vascular disease	1
Pulmonary embolism	4
Shortness of breath	4
Stroke	2
Dermatology	11
Contact Dermatitis	3
Foot swelling/pruritis	1
Lower Extremity Cellulitis	6
Retained Tick	1
Endocrine	2
Lightheadedness due to Hypothyroid	1
Fatigue	1
ENT	7
Acute otitis media	1
Contusion of nose	1
Epistaxis	1
Irritation of external ear canal	1
Seizures/vasovagal syncope	1
Vertigo	2
Gastrointestinal	47
Abdominal pain	7
Appendicitis	2
Chronic Cholecystitis	2
Chronic Diarrhea	1
Constipation	4
Dysphagia	6
Emesis	1
Esophageal varices	1
Esophagitis	4
GERD	2
Gastrointestinal Bleed	9
Hematochezia	4
Ileitis	1
Peptic ulcer	3
Immunology	10
Allergic Dermatitis	1
Full Body Hives	1
Idiopathic Angioedema	2
Neutropenia	1
Viral illness	5
Infectious Disease	7
Fever	5
Night Sweats	1
Sepsis	1
Neurology	10
Back Pain	4
Concussion	3
Multiple Sclerosis flare	1
Syncope and Seizures	2
Ophthalmology	4
Closed Fracture of Orbital Wall	1
Redness, Swelling of Eye	1
Vitreous detachment	2
Orthopaedic (not related to index surgery)	32
Calf Pain	2
Closed head Injury	3
Contusion of Hand	4
Hip Fracture	1
Lower back Pain	7
Lumbar laminectomy	1
Lysis of Adhesions from TKA	1
Patellar Fracture	1
Revision of Spinal Fusion	1
Shoulder Dislocation	1
Shoulder Pain	2
Trauma to toe	1
Subacromial Decompression	2
Vertebral Fracture	1
Wrist Fracture	4
Index Surgery Related	90
Infection/Surgical Site Drainage/I&D	27
Dislocation	6
Hip Pain	2
Implant Instability	4
Knee Pain	4
Knee Swelling	9
Lysis of Adhesions from TKA	24
Periprosthetic Fracture	6
Postoperative Anemia	3
Quadriceps Rupture	3
Stem Loosening	2
Psychiatry	3
Alcohol Withdrawal	1
Suicidal Ideation	2
Pulmonary	9
Asthma Exacerbation	2
Chest Pain with Shortness of Breath	3
COPD Exacerbation	2
Pneumonia	2
Renal	21
Acute kidney injury	2
Choledocholithiasis	2
Dysuria	1
Flank Pain	2
Hematuria	4
Hypokalemia	1
Pyelonephritis	4
Urinary Incontinence	3
Urinary Tract Infection (Lower)	2
Reproductive	4
Dysfunctional Uterine Bleeding	2
Spermatocele	1
Vaginal Laceration	1

Appendix C

**Table 10:** Patient Demographics of All-Cause Readmissions Within 90-Days Cohort CVA: Cerebrovascular Accident; CCI: Charlson Comorbidity Index; CHF: Congestive Heart Failure; SNF: Skilled Nursing Facility; BWH: Brigham and Women's Hospital; FH: Faulkner Hospital; MGH: Massachusetts General Hospital; NSMC: North Shore Medical Center; NWH: Newton-Wellesley Hospital; PROMIS: Patient-Reported Outcomes Measurement Information System; MCID: Minimal Clinically Important Difference; SF-10a: Short form-10a

	Unmatched			Matched		
Variables	No Readmissions (n=972)	Readmission in 90 days (n=102)	P-value	No Readmissions (n=102)	Readmission In 90 days (n=102)	P-value
Myocardial Infarction	13	1	0.762	0	1	0.316
Age	64.9	67.5	0.007*	68.1	67.5	0.611
Body Mass Index	29.8	30.2	0.543	30.6	30.2	0.595
Cancer	164	21	0.344	24	21	0.102
CVA	24	8	0.002*	9	8	0.800
CCI	0.9	1.0	0.813	1.3	1.0	0.136
CHF	16	1	0.608	2	1	0.561
Type II Diabetes	84	5	0.192	5	5	1.000
Discharge						
Home or Self Care	41	5	0.746	3	5	0.471
Home Healthcare	787	74	0.043*	80	74	0.329
Rehabilitation Facility	31	4	0.692	1	4	0.174
SNF	113	19	0.040*	18	19	0.856
Hospital						
BWH	272	24	0.338	25	24	0.870
FH	129	18	0.221	13	18	0.329
MGH	340	40	0.395	40	40	1.000
NSMC	61	8	0.539	9	8	0.800
NWH	170	12	0.143	15	12	0.535
Length of Stay	2.4	2.7	0.060	2.5	2.7	0.333
Male Sex	409	43	0.988	42	43	0.887
Total Knee Arthroplasty	531	70	0.007*	69	70	0.881
Preoperative PROMIS physical	42.3	42.1	0.842	42.3	42.1	0.839
Failure to Achieve 2-year PROMIS physical MCID	367	50	0.026*	42	50	0.260
Preoperative PROMIS mental	50.8	49.9	0.395	51.5	49.9	0.195
Failure to Achieve 2-year PROMIS mental MCID	591	69	0.177	60	69	0.191
Preoperative SF-10a	35.4	36.4	0.339	35.5	36.4	0.478
Failure to Achieve 2-year SF-10a MCID	410	56	0.014	49	56	0.327

Appendix D

**Table 11:** Patient Demographics of Non-index Surgery Related Readmissions Within 90-Days Cohort CVA: Cerebrovascular Accident; CCI: Charlson Comorbidity Index; CHF: Congestive Heart Failure; SNF: Skilled Nursing Facility; BWH: Brigham and Women's Hospital; FH: Faulkner Hospital; MGH: Massachusetts General Hospital; NSMC: North Shore Medical Center; NWH: Newton-Wellesley Hospital; PROMIS: Patient-Reported Outcomes Measurement Information System; MCID: Minimal Clinically Important Difference; SF-10a: Short form-10a

	Unmatched			Matched		
Variables	No Readmissions (n=972)	Readmission in 90 days (n=58)	P-value	No Readmissions (n=58)	Readmission In 90 days (n=58)	P-value
Myocardial Infarction	13	0	0.375	0	0	1.000
Age	64.9	68.0	0.011*	67.7	68.0	0.834
Body Mass Index	29.8	30.1	0.757	30.8	30.1	0.529
Cancer	164	15	0.079	14	15	0.830
CVA	24	4	0.044*	6	4	0.508
CCI	0.9	1.2	0.379	1.3	1.2	0.687
CHF	16	1	0.964	2	1	0.559
Type II Diabetes	84	2	0.165	7	2	0.083
Discharge						
Home or Self Care	41	2	0.776	1	2	0.559
Home Healthcare	787	39	0.011*	36	39	0.560
Rehabilitation Facility	31	1	0.532	0	1	0.315
SNF	113	16	0.001*	21	16	0.319
Hospital						
BWH	272	10	0.714	6	10	0.281
FH	129	10	0.075	10	10	1.000
MGH	340	26	0.128	31	26	0.353
NSMC	61	5	0.479	4	5	0.729
NWH	170	7	0.288	7	7	1.000
Length of Stay	2.4	2.8	0.028*	2.9	2.8	0.747
Male Sex	409	24	0.917	22	24	0.704
Total Knee Arthroplasty	531	39	0.061	37	39	0.696
Preoperative PROMIS physical	42.3	42.6	0.754	40.9	42.6	0.287
Failure to Achieve 2-year PROMIS physical MCID	367	31	0.017*	28	31	0.577
Preoperative PROMIS mental	50.8	49.8	0.462	50.1	49.8	0.882
Failure to Achieve 2-year PROMIS mental MCID	591	38	0.474	31	38	0.189
Preoperative SF-10a	35.4	37.3	0.165	35.5	37.3	0.260
Failure to Achieve 2-year SF-10a MCID	410	34	0.014	34	34	1.000

Appendix E

**Table 12:** Multivariable Regression of Matched 90-Day Non-orthopaedic Readmission Cohort to Predict Failure to Achieve A) Two-Year PROMIS-10 Physical, B) Two-Year PROMIS-10 Mental Health, and C) PROMIS Physical Function – Short Form 10a MCID With Significant Variables Reported (Matched) PROMIS: Patient-Reported Outcomes Measurement Information System; SF-10a: Short form-10a

Variable	Odds Ratio	95% Confidence Interval	P-value
Preoperative PROMIS physical	1.11	1.04-1.18	0.002
Variable	Odds Ratio	95% Confidence Interval	P-value
Preoperative PROMIS mental	1.12	1.06-1.20	<0.001
Variable	Odds Ratio	95% Confidence Interval	P-value
Total Knee Arthroplasty	2.83	1.11-7.68	0.034
Preoperative SF-10a	1.11	1.03-1.21	0.012
